# Lifetime Evaluation of Left Ventricular Structure and Function in Male C57BL/6J Mice after Gamma and Space-Type Radiation Exposure

**DOI:** 10.3390/ijms24065451

**Published:** 2023-03-13

**Authors:** Agnieszka Brojakowska, Cedric J. Jackson, Malik Bisserier, Mary K. Khlgatian, Cynthia Grano, Steve R. Blattnig, Shihong Zhang, Kenneth M. Fish, Vadim Chepurko, Elena Chepurko, Virginia Gillespie, Ying Dai, Brooke Lee, Venkata Naga Srikanth Garikipati, Lahouaria Hadri, Raj Kishore, David A. Goukassian

**Affiliations:** 1 Cardiovascular Research Institute, Icahn School of Medicine at Mount Sinai, New York, NY 10029, USA; 2 National Institute of Aerospace, Hampton, VA 23666, USA; 3 Department of Cell Biology and Anatomy and Physiology, New York Medical College, Valhalla, NY 10595, USA; 4 National Aeronautics and Space Administration, Hampton, VA 23669, USA; 5 Center for Comparative Medicine and Surgery, Icahn School of Medicine at Mount Sinai, New York, NY 10029, USA; 6 Department of Emergency Medicine, Dorothy M. Davis Heart Lung and Research Institute, The Ohio State University Wexner Medical Center, Columbus, OH 43210, USA; 7 Center of Excellence for Translational Medicine and Pharmacology, Department of Pharmacological Sciences, Icahn School of Medicine at Mount Sinai, New York, NY 10029, USA; 8 Department of Cardiovascular Sciences, Center for Translational Medicine, Lewis Katz School of Medicine, Temple University, Philadelphia, PA 19140, USA

**Keywords:** ionizing space radiation, cardiovascular disease, biomarkers, echocardiography, mathematical modeling

## Abstract

The lifetime effects of space irradiation (IR) on left ventricular (LV) function are unknown. The cardiac effects induced by space-type IR, specifically 5-ion simplified galactic cosmic ray simulation (simGCRsim), are yet to be discovered. Three-month-old, age-matched, male C57BL/6J mice were irradiated with ^137^Cs gamma (γ; 100, 200 cGy) and simGCRsim (50 and 100 cGy). LV function was assessed via transthoracic echocardiography at 14 and 28 days (early), and at 365, 440, and 660 (late) days post IR. We measured the endothelial function marker brain natriuretic peptide in plasma at three late timepoints. We assessed the mRNA expression of the genes involved in cardiac remodeling, fibrosis, inflammation, and calcium handling in LVs harvested at 660 days post IR. All IR groups had impaired global LV systolic function at 14, 28, and 365 days. At 660 days, 50 cGy simGCRsim-IR mice exhibited preserved LV systolic function with altered LV size and mass. At this timepoint, the simGCRsim-IR mice had elevated levels of cardiac fibrosis, inflammation, and hypertrophy markers *Tgfβ1*, *Mcp1*, *Mmp9*, and *βmhc*, suggesting that space-type IR may induce the cardiac remodeling processes that are commonly associated with diastolic dysfunction. IR groups showing statistical significance were modeled to calculate the Relative Biological Effectiveness (RBE) and Radiation Effects Ratio (RER). The observed dose-response shape did not indicate a lower threshold at these IR doses. A single full-body IR at doses of 100–200 cGy for γ-IR, and 50–100 cGy for simGCRsim-IR decreases the global LV systolic function in WT mice as early as 14 and 28 days after exposure, and at 660 days post IR. Interestingly, there is an intermediate time point (365 days) where the impairment in LV function is observed. These findings do not exclude the possibility of increased acute or degenerative cardiovascular disease risks at lower doses of space-type IR, and/or when combined with other space travel-associated stressors such as microgravity.

## 1. Introduction

Among the many genetic, lifestyle, and environmental stressors that affect the risk of developing cardiovascular disease (CVD), the leading cause of morbidity and mortality worldwide, ionizing radiation (IR) has emerged as a significant concern in some populations. Further, while radiation therapy (RT) has improved the overall survival in individuals with various malignancies, growing evidence shows that RT raises the risk of RT-induced CVDs, especially in patients receiving thoracic radiation near the heart [1,2,3]. For instance, in a population-based case-controlled study of 2168 women who underwent RT for breast cancer, the rates of major coronary events rose by 7.4% per gray (Gy) mean dose to the heart, with no apparent threshold. Incidence increased as early as five years post IR, and continued for three decades [1]. In addition, accelerated coronary atherosclerosis, pericardial pathologies, valvular diseases, and diastolic heart failure are other known RT-induced CVDs (reviewed in [4]).

With recent advances in space exploration and planned exploratory space missions, the risk of IR-induced CVD will become more concerning for astronauts on long-duration missions to the Moon and Mars. Beyond the protective magnetosphere in low-Earth orbit (LEO), where most astronauts remain during space flight on the International Space Station (ISS), crews will be exposed to higher cumulative doses of ionizing space IR. The space-IR environment beyond LEO predominantly comprises solar energetic particles (SEPs) and galactic cosmic rays (GCRs). The sun sporadically emits SEPs as large plasma clouds composed primarily of low–medium-energy protons (^1^H) (i.e., solar particle events (SPE)), which can be effectively shielded. GCRs originate from outside the solar system and contain high-energy protons (90%), helium particles (9%), and HZE (high charge (H), atomic number (Z), and energy (E)) nuclei (1%) [5]. HZE nuclei have a high linear energy transfer (LET) which can penetrate spacecraft shielding and produce densely ionizing tracks that can cause clustered DNA damage that is difficult for cells to repair. These particle tracks also emanate delta rays that further damage cells and that even reach neighboring cells. The biological effects of these HZE nuclei may pose considerable IR-induced CVD risks, but they are not yet well characterized [5].

One of the challenges in estimating space IR-induced CVD risks for astronauts on deep-space missions is the lack of relevant human data. Few humans have been exposed to space IR beyond LEO, and for those who have, spaceflight time and cumulative IR doses were much lower than what is estimated for long-duration missions to the Moon and Mars. To help address this knowledge gap, we will characterize cardiovascular (CV) responses to space IR using animal models, and the comparative effects of the radiation types will be calculated using the Relative Biological Effectiveness (RBE) and Radiation Effects Ratio (RER) [6].

Work is already underway using animal models to determine space IR-induced CVD risk. Our prior research has shown that p-H2AX foci, a well-described marker of double-strand DNA breaks (DSB), were increased 2–5-fold in C57B1/6NT mouse cardiomyocytes (CMs) and endothelial cells (ECs) 2–24 h after whole-body exposure to 50 cGy ^1^H (1 GeV; LET = 0.223 keV/µm) or 15 cGy ^56^Fe (1 GeV/n; LET = 151.4 keV/µm). More importantly, p-H2AX foci remained above the background level in the CMs of mice exposed to ^56^Fe IR at 28 days post IR, suggesting that p-H2AX foci decay significantly slower in CMs versus ECs. By 3 and 10 months post IR, ^56^Fe IR mice had expanded myocardial fibrosis, altered cardiac function, and impaired recovery after myocardial infarction (MI) [7]. We have also demonstrated that different sequences of whole-body, fractionated, single, low-dose ion [^1^H, ^56^Fe] IR induced markedly different biological responses under normal aging conditions and after induced (via left anterior descending artery (LAD) ligation) MI [8]. Further, Tungjai et al. have confirmed that 10–12-week-old male CBA/CaJ mice exposed to fractionated ^28^Si IR (0.1–0.5 Gy, 300 MeV/n) exhibited more apoptosis and chronically activated NF-κB and related pro-inflammatory cytokines (IL-1β, IL-6, TNFα) up to six months post IR [9]. A study by Garikipati et al. showed altered cardiac gene expression 16 months post IR in female CB6F1/Hsd mice exposed once to one of four different doses of either γ ^137^Cs (40–160 cGy, 0.662 MeV), silicon (^14^Si) (4–32 cGy, 260 MeV/n), or titanium (^22^Ti) (3–26 cGy, 1 GeV/n) IR. No clear lower IR threshold was determined; however, we identified that differentially expressed genes were involved in several CVDs, including cardiomyopathies, myocardial injury, and cardiac hypertrophy [10]. Additional studies are still needed to examine the effects of varying IR doses, dose rates, and mixed HZE fields in order to estimate their comparative impacts on the CVD risk associated with long-duration deep-space missions.

Continuing these efforts, we longitudinally assessed in detail the effects of gamma (γ) and simplified galactic cosmic ray simulation (simGCRsim) IR on cardiac left ventricular (LV) function and structure, using transthoracic echocardiography in male C57BL/6J (wild type (WT)) mice. Transthoracic echocardiography is a standard non-invasive diagnostic tool that comprehensively assesses cardiac structure and function. The ability of the heart to meet systemic metabolic demands is reflected in cardiac output (CO), which reflects the volume of oxygenated blood distributed into systemic circulation during systole (contraction) [11]. CO is dependent primarily on stroke volume (SV), a measure of the volume ejected from the LV during systole (i.e., the change in blood volume in the LV from diastole (relaxation) to systole), and heart rate [12]. Three parameters dictate SV: preload, contractility, and afterload. Echocardiography provides a non-invasive method to qualitatively and quantitively assess the heart’s function for each parameter. Preload refers to the degree of myocardial stretch accompanied by venous return (ventricular filling), which is reflected by the end diastolic volume (EDV). Outside of the venous return, the amount of blood remaining within the LV after ventricular ejection (end systolic volume; ESV) is also dependent on the contractility of the myocardium and is reflected by the LV ejection fraction (LVEF). LVEF reflects the systolic (contractile) function and is a fundamental parameter in assessing cardiac function and determining whether an impaired systolic or diastolic function is dictating a given pathophysiology and clinical presentation. While systolic function focuses on contractility, diastolic function refers to the ability of the LV to relax and to comply with holding an increasing blood volume during filing. Finally, afterload refers to the resistance that needs to be overcome with blood ejection from the LV, including pathologies such as aortic stenosis or upstream aortic atherosclerosis, which obstructs blood flow out of the LV into the aorta. In various scenarios where there is a pathologic disturbance of these components (whether an inherent cardiac or systemic cause), echocardiography also provides an opportunity to assess compensatory structural changes such as hypertrophy and/or the atrophy of ventricular walls in response to altered hemodynamics [13,14]. Overall, echocardiography offers a critical modality for evaluating cardiac function and identifying subclinical changes in cardiac function and structure which may reflect underlying disease processes.

The aims of this study were to (1) determine the effects of γ (terrestrial) and simGCRsim (space) IR on LV function; (2) identify disease spectrum and latency following single, whole-body IR exposure; (3) establish whether dose thresholds for γ and simGCRsim IR exist; and (4) describe the comparative impact of γ and simGCRsim IR by calculating RBEs and RERs. To address these objectives, we irradiated three-month-old, age-matched male WT mice with γ (100, 200 cGy) and simGCRsim (50, 100 cGy) IR. We tracked the longitudinal effects of IR on LV function using echocardiography performed at two acute (14, 28 days post IR) and three long-term—i.e., degenerative—(365, 440, and 660 days post IR) time points. RBE and RER estimates were calculated for LV ejection fraction and LV fractional shortening, on which IR had a statistically significant effect. We hypothesized that γ and simGCRsim IR would induce chronic, IR type-dependent biological responses that may increase the relative risk of developing CVD.

## 2. Results

### 2.1. Effects of Irradiation on Physical Parameters in Mice

**Figure 1:**
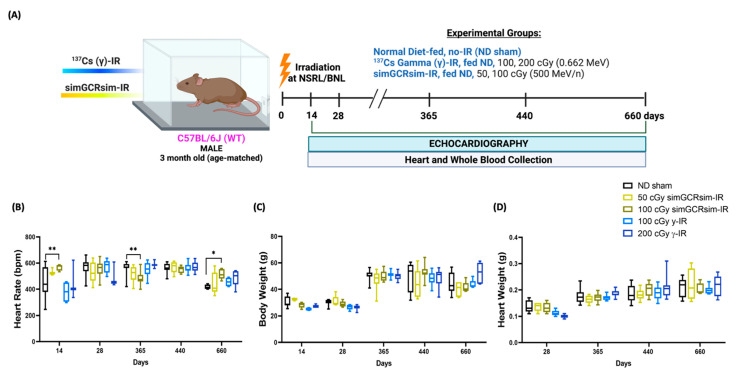
Physiological response of C57BL/6J male mice to gamma and simGCRsim-IR. (**A**) Three-month-old age-matched C57BL/6J wild-type mice were irradiated with ^137^Cs-γ-IR (100, 200 cGy) and simGCRsim (50, 100 cGy). We assessed LV function via transthoracic echocardiography at 14, 28, 365, 440, and 660 days post IR. No-IR normal diet (ND)-fed mice served as negative control. In addition to transthoracic echocardiography, blood and hearts were collected at each time point. Created with BioRender.com. (**B**) Heart rates were obtained during transthoracic echocardiography, while mice were anesthetized using isoflurane (3–4% induction, 1–2% maintenance). (**C**) Animal bodyweights. (**D**) Heart weights post exsanguination. For acute time points (14 and 28 days), *n* = 3–10 animals (50 cGy simGCRsim and 200 cGy gamma-IR at 14 days, *n* = 3; the rest of the samples for 14 and 28 days, *n* = 5–10). For the degenerative time points (365, 440, and 660 days), *n*= 10–15 animals. *p*-values were calculated using two-way ANOVA, ** *p* < 0.01, * *p* < 0.05. Note, due to technical reasons, heart weights were not collected at the 14-day timepoint.

We assessed irradiation effects on mouse development at each stage of the experimental protocol (Figure 1A). Compared to the control normal diet (ND) sham-IR mice, 100 cGy simGCRsim-IR mice had significantly higher heart rates at 14 days (*p* = 0.002) and 660 days (*p* = 0.02) post IR, but lower at 365 days (*p* = 0.007) during image acquisition (Figure 1B). Echocardiography showed no significant difference in the heart rate between the remaining treatment groups at any collection time point (Figure 1B). Additionally, neither the body weight nor the heart weight differed significantly between both the IR groups compared to the ND sham (Figure 1C,D). With regard to the overall mortality, there were more spontaneous deaths and physical conditions requiring euthanasia, starting at 440 days post IR in 100 cGy simGCRsim-IR and γ-IR mice compared to ND sham (Supplementary Figure S1). Interestingly, there were fewer spontaneous deaths in 50 cGy simGCRsim-IR compared to ND sham, possibly due to the relatively higher rates of infection/sepsis in ND sham mice (Supplementary Table S1). Necropsies revealed no definitive cause of death in irradiated animals; however, there was evidence of myocardial and vascular injury and cancer in some low-dose γ-IR animals and No-IR sham mice (Supplementary Table S1).

### 2.2. Acutely Altered LV Function following Irradiation

**Figure 2:**
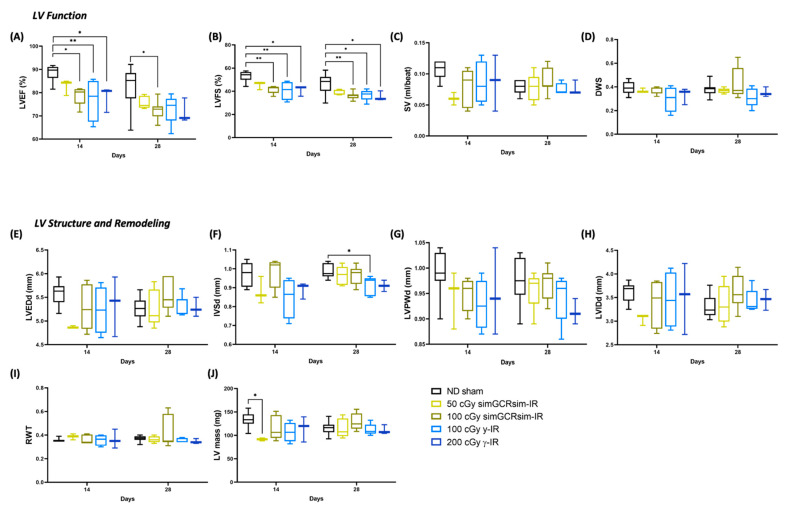
Longitudinal echocardiography results in C57BL/6J male mice at 14 and 28 days after gamma (γ) and simGCRsim irradiation. Three-month-old male C57BL/6J mice were irradiated with simGCRsim-IR (50 and 100 cGy) or gamma (γ)-IR (100 and 200 cGy). Left ventricular (LV) function is represented by (**A**) ejection fraction, (**B**) fractional shortening, (**C**) stroke volume (SV), and (**D**) radial wall strain (DWS). Parameters of LV dimensions and remodeling are represented by (**E**) LV end diastolic diameter, (**F**) intraventricular septal thickness, (**G**) LV posterior wall thickness, (**H**) LV internal cavity diameter, (**I**) relative wall thickness, and (**J**) LV mass. No-IR normal-diet (ND)-fed mice served as controls. *n* = 3–10 mice per treatment condition and time point (50 cGy simGCRsim and 200 cGy gamma-IR at 14 days, *n* = 3; the rest of samples for 14 and 28 days, *n* = 5–10); *p*-values were calculated using one-way ANOVA. ** *p* < 0.01, * *p* < 0.05.

To determine the acute “in-flight” effects space-type (simGCRsim) and γ-IR have on LV function, echocardiography was performed at 14 and 28 days post IR (Figure 2). These time points serve as in-flight approximations, as ~1 month of mouse life equals 2.9 years of human life, which is also the time estimate for future Mars exploration missions. By 14 days post IR, global LV systolic function, as indicated via LV ejection fraction (LVEF) and LV fractional shortening (LVFS), decreased in 100 cGy simGCRsim and 100 and 200 cGy of g-IR groups (Figure 2A,B). Although there was a small, not statistically significant drop in LVEF and LVFS in 50 cGy simGCRsim versus ND sham mice at 14 days post IR, the stroke volume (SV) was ~50% lower (0.06 mL/beat ± 0.006 vs. ND sham 0.11 mL/beat ± 0.005, approaching significance, *p* < 0.08), the LV end-diastolic dimension (LVEDd) was 12.5% lower (4.9 mm ± 0.02 vs. ND sham 5.6 mm ± 0.08, approaching significance, *p* < 0.059), and the LV mass was ~30% lower (91 mg ± 1.8 vs. ND sham 133 mg ± 5.2, significant, *p* < 0.03), all of which may suggest possible cardiac decompensation at this early (“in-flight”), post IR time point (Figure 2A–C,E–H,J). Compared to the ND sham mice, 50 cGy simGCRsim-IR mice still had reduced global LV systolic functions (LVEF and LVFS) 28 days post IR, but neither LV structure nor mass were considerably altered (Figure 2A,B,E–J). This suggests that, by this time, the mice had begun to compensate for the acute systolic impairment.

In mice irradiated with 100 cGy simGCRsim, global LV systolic function was significantly diminished at both acute time points, as reflected by a ~10% decrease in both LVEF (14 days: 79% ± 1.8 vs. ND sham 89% ± 1.2, *p* < 0.02; 28 days: 72% ± 1.2 vs. ND sham 83% ± 2.7, *p* < 0.02) and LVFS (14 days: 42% ± 1.6 vs. ND sham 53% ± 1.6, *p* = 0.004; 28 days: 37% ± 1.2 vs. ND sham 46% ± 2.6, *p* < 0.01) (Figure 2A,B). No significant structural alterations were noted in 100 cGy simGCRsim-IR mice (Figure 2E–J).

At both γ-IR doses, the global LV systolic function was significantly abated at 14 days post IR, with ~10% lower LVEF (100 cGy 77% ± 4.6 and 200 cGy 78% ± 3.1 vs. ND sham 89% ± 1.2, *p* < 0.05) and LVFS (100 cGy 41% ± 4.0 and 200 cGy 41% ± 2.6 vs. ND sham 53% ± 1.6, *p* < 0.05 (Figure 2A,B). Although not statistically significant, at 14 days, the interventricular septal thickness in diastole (IVSd) dropped 20% in 100 cGy γ-IR mice (IVSd; 0.8 mm ± 0.05 vs. ND sham 1 mm ± 0.02, approaching significance, *p* < 0.055) (Figure 2F). The global LV systolic function remained decreased at 28 days post IR in 100 cGy γ-IR mice compared to ND sham (LVEF: 100 cGy 73% ± 2.8 vs. ND sham 83% ± 2.7, *p* = 0.05; LVFS: 100 cGy 37% ± 2.1 vs. ND sham 47% ± 2.6, *p* < 0.03), a result that was associated with significantly reduced IVSd (0.91 mm ± 0.02 vs. ND sham 0.99 mm ± 0.01, *p* < 0.02) (Figure 2A,B,F). In 200 cGy γ-IR mice, LVEF remained reduced at 28 days post IR (72% ± 3.0 vs. ND sham 83% ± 2.7, approaching significance, *p* < 0.08) and was associated with significantly lessened LVFS (36% ± 2.4 vs. ND sham 47% ± 2.6, *p* < 0.05) (Figure 2A,B). No significant alterations in radial wall contractility (DWS), relative wall thickness (RWT), or LV internal diameter (LVIDd) were observed among all treatment conditions at these early timepoints (Figure 2D,H,I).

### 2.3. Degeneratively Altered LV Function following Irradiation

**Figure 3:**
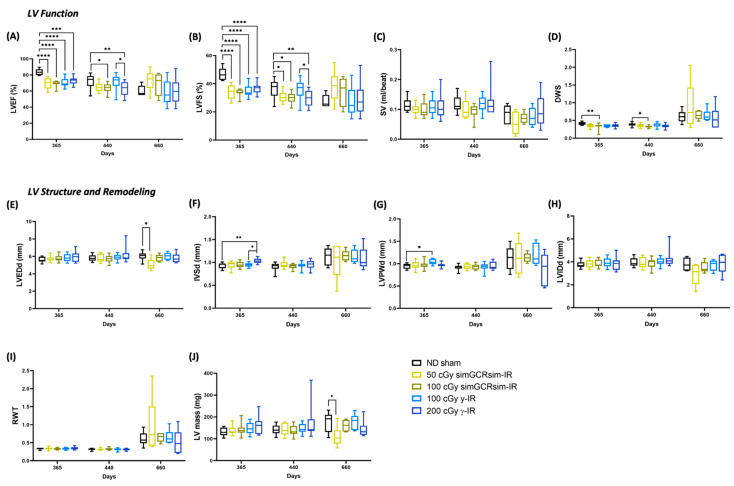
Longitudinal echocardiography results in C57BL/6J male mice at 365, 440, and 660 days after gamma (γ) and simGCRsim irradiation. Left ventricular (LV) function is represented by (**A**) ejection fraction, (**B**) fractional shortening, (**C**) stroke volume (SV), and (**D**) radial wall strain (DWS). Parameters of LV dimensions and remodeling are represented by (**E**) LV end diastolic diameter, (**F**) intraventricular septal thickness, (**G**) LV posterior wall thickness, (**H**) LV internal cavity diameter, (**I**) relative wall thickness, and (**J**) LV mass. *n* = 10–15 mice per treatment condition and time point; p-values were calculated using one-way ANOVA. **** *p* < 0.0001, ** *p* < 0.01, * *p* < 0.05.

We performed echocardiography at 365, 440, and 660 days post IR to assess the long-term (degenerative) effects of simGCRsim and γ-IR on LV function (Figure 3). By 365 days post IR, all IR treatment groups exhibited significantly reduced LV systolic function (*p* < 0.0001) (Figure 3A,B). Compared to the ND sham mice, mice irradiated with either simGCRsim dose had significantly decreased (by 13–15%) LV systolic function (LVEF: 50 cGy 69% ± 2.1 and 100 cGy 69% ± 1.1 vs. ND sham 83% ± 1.3, *p* < 0.0001; LVFS: 50 cGy 34% ± 1.6 and 100 cGy 34% ± 0.7 vs. sham 47% ± 1.5, *p* < 0.0001). In 100 cGy simGCRsim-IR mice, this was associated with a significantly lower diastolic wall strain (DWS), a parameter that reflects LV resistance to diastolic deformation (wall thinning) based on elastic theory [15,16], suggesting these mice have increased LV myocardial stiffness (0.32 ± 0.03 vs. ND sham 0.42 ± 0.01, *p* < 0.008) (Figure 3A,B,D). Although not statistically significant (but approaching significance), the DWS also dropped in mice irradiated with lower-dose simGCRsim (0.35 ± 0.02 vs. ND sham 0.42 ± 0.01, approaching significance, *p* = 0.059), indicating that space-type IR may promote LV myocardial stiffness through remodeling processes that may compromise LV compliance (Figure 3A,B,D). We observed no significant structural alteration in 50 or 100 cGy simGCRsim-IR mice at this time point (Figure 3E–J). Compared to the ND sham mice, both LVEF and LVFS were significantly reduced in mice irradiated with either dose of γ-IR (LVEF: 100 cGy 70% ± 1.8 and 200 cGy 73% ± 1.6 vs. ND sham 83% ± 1.3, *p* < 0.001; LVFS: 100 cGy 35% ± 1.4 and 200 cGy 37% ± 1.3 vs. ND sham 47% ± 1.5, *p* < 0.0001) (Figure 3A,B). In 100 cGy γ-IR, this was associated with a significantly expanded LV posterior wall thickness (LVPWd: 1.04 mm ± 0.02 vs. ND sham 0.94 ± 0.02, *p* < 0.01) and >15% less DWS (0.35 ± 0.01 vs. ND sham 0.42 ± 0.01, approaching significance, *p* < 0.08) (Figure 3D,G). Yet, IVS was significantly thicker in 200 cGy γ-IR mice compared to both lower-dose γ-IR and ND sham mice (1.0 mm ± 0.01 and 100 cGy γ 0.94 mm ± 0.01, *p* < 0.01; vs. ND sham 0.93 mm ± 0.02, *p* < 0.002) (Figure 3F). This suggests that γ-IR mice may be compensating for a reduced systolic function with hypertrophy.

At 440 days, the global LV systolic function remained impaired in mice irradiated with either dose of simGCRsim-IR (Figure 3A,B). In 50 cGy simGCRsim-IR mice, LVFS was significantly reduced by ~6% (31% ± 1.1 vs. ND sham 37% ± 1.6, *p* < 0.04), and LVEF by 7% (65% ± 1.5 vs. ND sham 72% ± 2.1, approaching significance, *p* = 0.07) (Figure 3A,B). No changes in cardiac structure were noted in 50 cGy simGCRsim-IR mice at this time. Like the LV systolic function (LVEF: 64% ± 1.9 vs. ND sham 72 ± 2.1, *p* < 0.02; LVFS: 30% ± 1.3 vs. ND sham 37% ± 1.6, *p* < 0.01), DWS was also significantly decreased in 100 cGy simGCRsim-IR mice, suggesting persistent LV myocardial stiffness (0.33 ± 0.01 vs. ND sham 0.39 ± 0.01, *p* < 0.01) (Figure 3A,B,D). LV structure and function did not significantly differ between 100 cGy γ-IR mice and ND sham at 440 days; however, the global LV systolic function was significantly impaired in 200 cGy γ-IR mice compared to both 100 cGy γ-IR and ND sham (LVEF: 62% ± 2.4 vs. ND sham 72% ± 2.1, *p* < 0.01; LVFS: 30% ± 1.6 vs. ND sham 37% ± 1.6, *p* < 0.01) (Figure 3A,B).

The effects of IR on systolic function appear to be stable at 660 days post IR for all radiation groups, demonstrating that this may be when the impacts of aging and IR intersect (Figure 3A,B). Mice irradiated with either γ-IR dose (100 or 200 cGy) had no significant alterations in LV function or structure, compared to ND sham mice, with an average LVEF of 58% (Figure 3). Although not statistically significant, both 50 and 100 cGy simGCRsim-IR mice showed preserved LVEF (50 cGy 73% ± 5.3 and 100 cGy 68% ± 6.3 vs. ND sham 60% ± 2.7) and LVFS (50 cGy 38% ± 4.5 vs. 100 cGy 34% ± 4.7 vs. ND sham 28% ± 1.8) compared to ND sham (Figure 3A,B). However, in 50 cGy simGCRsim mice, preserved LVEF is associated with a significantly decreased overall LV size compared to ND sham (LVEDd: 5 mm ± 0.3 vs. ND sham 6 mm ± 0.2, *p* < 0.02), likely due to a smaller overall LV cavity size (LVIDd: 2.9 mm ± 0.4 vs. ND sham 3.8 mm ± 0.2, approaching significance, *p* < 0.059) and ~37% less LV mass (110 mg ± 18.6 vs. ND sham 176 mg ± 18.9, *p <* 0.05) (Figure 3E,H,J), changes that suggest cardiac decompensation may be in progress. Additionally, relative wall thickness (RWT), a marker of LV remodeling, was abnormally high in a large percentile of mice irradiated with low-dose simGCRsim-IR, a result that indicates possible cardiac hypertrophy (Figure 3I). Overall, we observed no significant, sustained dose-response in either IR group across all the examined timepoints, thereby demonstrating that the upper threshold for degenerative cardiac responses may have been saturated at lower doses of either IR. Note that substantial inter-animal variability in cardiac responses occurred for many LV structural parameters across all time points.

### 2.4. Perivascular Fibrosis in LV

**Figure 4:**
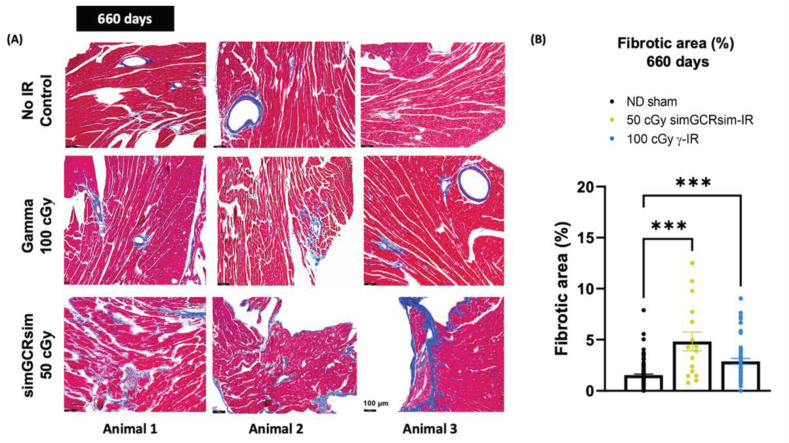
Myocardial fibrosis at 660 days post IR. Transverse sections of hearts at basal and midventricular levels (four heart tissue sections taken 80 µm apart from each other) were stained with Masson’s Trichrome to assess fibrotic burden. (**A**) Representative images of heart sections from three different animals show fibrosis, highlighted in bright blue, in contrast with dark, red-stained myocardium. Scale 100 µm, 200× magnification. (**B**) Fibrosis burden in heart tissue was quantified using the ImageJ program, with the fraction of the blue stained area being normalized to the total red area. *n* = 3 mice per treatment condition; *p*-values were calculated using one-way ANOVA. *** *p* < 0.001.

Considering that the substantial structural differences (LV mass, LVIDd, IVS) noted between IR groups appear at 660 days, and that no significant sustained dose response was observed, the cross-sections of hearts collected at 660 days post IR from ND sham, 50 cGy simGCRsim-IR, and 100 cGy γ-IR mice were stained with Masson’s Trichrome to assess the fibrotic burden (Figure 4A). Compared to the ND sham mice, γ-IR and simGCRsim-IR had a significant 2–3-fold increase (*p* < 0.0001) in LV perivascular and interstitial fibrosis at 660 days (Figure 4A,B).

### 2.5. Expression of Markers of Cardiac Hypertrophy, Fibrosis, and Inflammation

**Figure 5:**
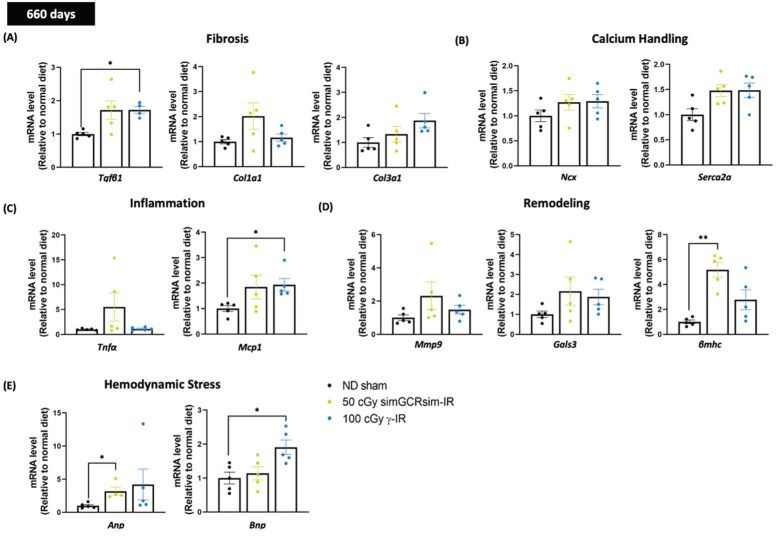
Gene expression in left ventricular tissue was assessed 660 days after simGCRsim and gamma (γ) irradiation. Relative mRNA expression of (**A**) cardiac fibrosis (*Tgfβ1*, *Col1a1*, and *Col3a1*), (**B**) calcium handling (*Ncx*, and *Serca2a*), (**C**) inflammation (*TNFα*, and *MCP1*), (**D**) cardiac remodeling (*Mmp9*, *Gals3*, and *βmhc*), and (**E**) hemodynamic stress (*Anp*, and *Bnp*) markers within the left ventricle (LV) of mice irradiated with 50 cGy simGCRsim or 100 cGy γ at 660 days post-irradiation. *Tgfβ1* indicates transforming growth factor beta 1; *Col1a1*, type I fibrillar collagen; *Col3a*, type III fibrillar collagen; *Ncx*, cardiac sodium-calcium exchanger; *Serca2a*, Sarcoplasmic Reticulum Ca^2+^ ATPase; *Tnfα*, tumor necrosis factor alpha; *Mcp1*, monocyte chemoattractant protein-1; *Mmp9*, matrix metalloproteinase-9; *Gals3*, galectin 3; *βmhc*, cardiac beta myosin heavy chain; *Anp*, atrial natriuretic peptide; *Bnp*, brain natriuretic peptide. Each dot represents an individual mouse. Expression was normalized to *GAPDH*. Data reported as mean ± SEM. *n* = 5 animals/treatment group; *p*-values were calculated using Kruskal-Wallis *H* test, ** *p* < 0.01, * *p* < 0.05.

To determine the underlying mechanisms that may be responsible for the LV functional changes observed in both IR groups, we evaluated the expression of genes involved in the cardiac remodeling processes in the LV of ND sham, 50 cGy simGCRsim-, and 100 cGy γ-IR mice at 660 days post IR via qPCR (Figure 5).

Transcript levels of transforming growth factor-β1 (*Tgfβ1*), crucial for inducing and maintaining cardiac fibroblast activation and collagen synthesis during cardiac fibrosis [17], were elevated (1.7-fold higher) in 100 cGy γ-IR mice compared to ND sham (*p* < 0.03) (Figure 5A). A similar trend (approaching significance, *p* < 0.08) was observed in 50 cGy simGCRsim-IR mice, suggesting that fibrotic remodeling via *Tgfβ1* may be a common pathway between terrestrial γ- and space-type IR. Although not statistically significant, the mRNA levels of type I (*Col1a1*) and type III fibrillar collagen (*Col3a1*) were increased in simGCRsim and γ-IR compared to ND sham, a result that indicates that collagen remodeling may be specific for both IR types (Figure 5A).

Additionally, the transcript levels of Sarcoplasmic Reticulum Ca^2+^ ATPase (*Serca2a*), which is critical in mediating cardiomyocyte contraction and sarcoplasmic reticulum calcium re-uptake [18], were also augmented in both 50 cGy simGCRsim-IR and 100 γ-IR (approaching significance in both, *p* = 0.08 and *p* = 0.056, respectively) than in ND sham (Figure 5B), which shows the heart may be compensating to preserve cardiac function at 660 days post IR. Sodium-calcium exchanger (*Ncx*) did not significantly differ in either IR group. 

Regarding inflammation, there was no notable variation in tumor necrosis alpha (*Tnfα*) expression in either IR group. However, the levels of monocyte chemoattractant protein (*Mcp1*), a chemokine key to regulating monocyte/macrophage migration and infiltration [19], were significantly elevated in the LV of 100 cGy γ-IR mice compared to ND sham (*p* < 0.03), suggesting that γ-IR may promote cardiac inflammation (Figure 5C). In 50 cGy simGCRsim-IR mice, the mRNA levels of beta-myosin heavy chain (*βmhc*), which is a marker for cardiac hypertrophy and which may contribute to maladaptive responses in cardiovascular stress [20], were significantly augmented compared to ND sham (*p* < 0.003), thereby indicating that simGCRsim may promote hypertrophic remodeling that could exacerbate impaired cardiac contractility (Figure 5D). There was no significant difference in either the *Mmp9* (matrix metalloproteinase 9, involved in degrading cardiac extracellular matrix components) or *Gals3* (galectin-3, a regulatory β-galactoside-binding lectin that participates in cardiac fibrosis) levels, though both IR groups had higher overall levels than ND sham (Figure 5D). In addition to assessing the genes involved in cardiac remodeling, we evaluated the transcript levels of two known heart failure markers—atrial natriuretic peptide (*Anp*) and brain natriuretic peptide (*Bnp*) [21]—in the LV of mice 660 days post IR (Figure 5E). In 50 cGy simGCRsim-IR mice, *Anp* levels were significantly higher compared to ND sham (*p* < 0.02) (Figure 5E). Along with a trend toward elevated *Anp* levels (*p* = ns), BNP levels were significantly increased in 100 cGy γ-IR mice (*p* < 0.03) (Figure 5E). This suggests that mice in both IR groups are both exhibiting and responding to some form of cardiac stress (hypervolemia, pressure overload).

### 2.6. Circulating Cardiac Markers of Hemodynamic Stress and Endothelial Dysfunction

**Figure 6:**
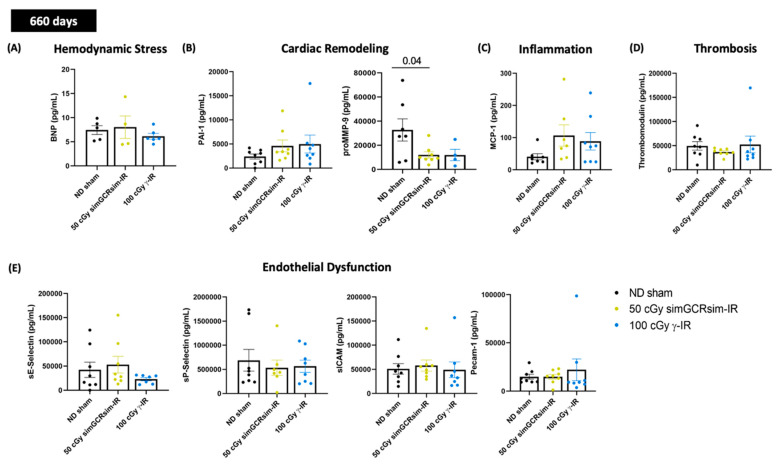
Select cardiac serum markers assessed 660 days after gamma and simGCRsim irradiation. Peripheral blood serum was processed using standard ELISA to assess serum levels of (**A**) brain natriuretic peptide (BNP). Remaining samples were used for Multiplex assay to assess markers of (**B**) cardiac remodeling: plasminogen activator inhibitor-1 (PAI-1) and pro-matrix metalloprotease-9 (proMMP-9), (**C**) inflammation: Monocyte Chemoattractant Protein-1 (MCP-1), (**D**) Thrombosis: thrombomodulin, and (**E**) endothelial dysfunction: sE-Selectin, sP-Selectin, sICAM, and PECAM-1. All values are reported as pg/mL as mean ± SEM. *n* = 5 animals/treatment group. *p*-values were calculated using Kruskal-Wallis *H* test.

To determine whether known cardiac biomarkers of hemodynamic stress and vascular function accompany the observed LV alterations, the serum levels of select biomarkers were processed from ND sham, 50 cGy simGCRsim-IR, and 100 cGy γ-IR mice at all degenerative time points (660 days post IR, Figure 6A–E; 360 and 440 days, Supplementary Figure S2). Across all timepoints, there were no significant differences in the serum levels of BNP, a marker for heart failure, in either IR group (Figure 6A and Supplementary Figure S2A). Serum biomarkers were not significantly changed at 365 days post IR in either IR group compared to ND sham (Supplementary Figure S2A–E). Interestingly, at 440 days, only serum pro-matrix metalloproteinase-9 (proMMP-9), which is involved in LV remodeling and cardiac repair, was 3–4-fold lower (*p* < 0.05) in simGCRsim-IR than in ND sham and 100 cGy γ-IR mice (Supplementary Figure S2B). Additionally, the serum levels of platelet endothelial cell adhesion molecule 1 (PECAM-1), which rises following myocardial infarctions and thrombotic events [22,23], were also significantly lower at 440 days (*p* = 0.009) (Supplementary Figure S2E). 

At 660 days post IR, serum plasminogen activator inhibitor-1 (PAI-1), confirmed to be antifibrotic through prior murine studies [24,25], was expanded in both IR groups (simGCRsim-IR 4605 pg/mL ± 3.460 and γ-IR 4982 pg/mL ± 5.276 vs. ND sham 2389 pg/mL ± 1.489) (Figure 6B). Though this rise is not statistically significant, it is worth noting, particularly considering the very high rates of inter-animal variability. Interestingly, serum proMMP-9 was substantially lower in both simGCRsim-IR (2.3-fold lower, *p* > 0.04) and γ-IR mice (3.6-fold lower, approaching significance, *p* = 0.07) compared to ND sham, a finding that suggests that after IR, certain cardiac compensatory mechanisms may be either impaired or temporally regulated, depending on the IR type (Figure 6B). Serum levels of MCP-1 at 660 days showed a >2-fold elevation trend (approaching significance, *p* = 0.06) in both simGCRsim (106.7 pg/mL ± 33) and γ-IR (88.7 pg/mL ± 27) mice compared to ND-sham (40.60 pg/mL ± 9), indicating that IR and aging may accelerate inflammation, which may drive the remodeling processes (Figure 6C). No significant alterations in the serum markers of endothelial dysfunction/inflammation (spSelectin, seSelectin, PECAM-1, sICAM) or thrombosis (Thrombomodulin) were observed at 660 days post IR (Figure 6D,E).

### 2.7. BMD Dose-Response Modeling

**Figure 7:**
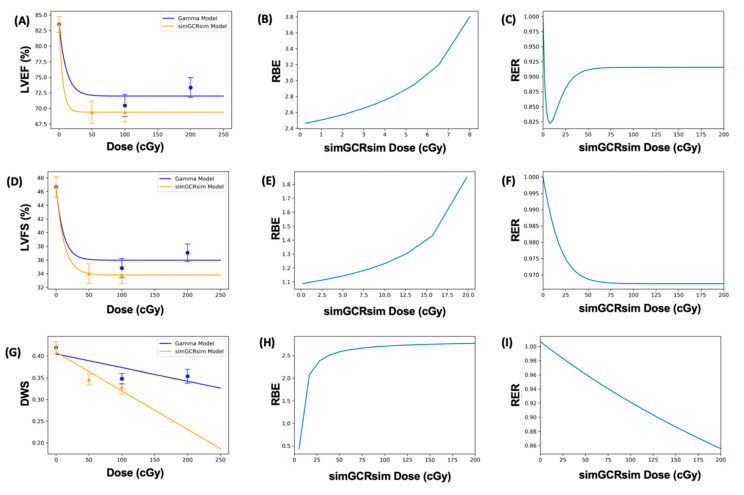
Modeling and comparative effect measures of C57Bl/6J mice 365 days after exposure to gamma and simGCRsim irradiation. Comparison of (**A**) LVEF, (**D**) LVFS, and (**G**) DWS mean dose-response data (SEM error bars) superimposed on BMD dose-response models for gamma (orange triangles) and simGCRsim (blue circles) irradiation. To the right are the corresponding RBE calculations within the viable ranges (**B**,**E**,**H**), and the corresponding RER calculation (**C**,**F**,**I**) for LVEF, LVFS, and DWS, respectively.

Regarding the cardiac response to dose, LVEF and LVFS showed an initial drop that then flattened out. This dose-response shape was best fit using the Frequentist Exponential degree 4 (Freq. Exp. Deg. 4) model (example shown in Figure 7A,D,G). These models reported the Benchmark Dose (BMD), a dose level that indicates where the risk rises above the background by some predefined level, which was very low or near zero. The BMD estimates a threshold dose for radiation response. Yet, because the BMD values were so low, any existing threshold would be below the lowest measured radiation dose (50 cGy simGCRsim or 100 cGy γ) for LVEF and LVFS. The DWS dose-response was best fit using a linear model, which does not exhibit a threshold (an example is shown in Figure 7G).

### 2.8. Dose-Response Models with Dichotomized Data

When the data were dichotomized (normal: LVEF 50–100%, LVFS 40–100%; diseased: LVEF < 50%, LVFS < 40%) and modeled in BMDs, the results were considerably uncertain (as expressed by the large error bars that represent 95% CI on the proportions observed) due to large individual variation, and small sample sizes. For LVFS, the substantial uncertainty was also influenced by the relatively large percentage of individuals who fell within the “diseased” category. This uncertainty rendered the dichotomized data unsuitable for dose-response modeling (Figure 7D). For LVEF, there were very few instances where an individual fell within the “diseased” range. This lack of a strong response meant that the LVEF data were also not appropriate for dichotomized dose-response modeling (Figure 7A).

### 2.9. RBE and RER Calculations

The Relative Biological Effectiveness (RBE) is the ratio between the γ- and simGCRsim-IR doses that induce the same biological response. RBE was undefined over much of the dose range [6]. More specifically, the interval for which an RBE could be calculated ranged from 8.02 cGy to 107.95 cGy for LVEF, and from 19.84 cGy to 104.34 cGy for LVFS (Table 1 and Figure 7B,E). For LVFS, the Freq. Exp. Deg. 4 model gave a more linear fit to the day 440 γ-IR data, and allowed the RBE to be calculated for the entire simGCRsim dose range (Figure 7E). For DWS, the linear dose-response model best fits the data and allowed the RBE to be defined along the entire range of simGCRsim IR doses measured (Table 1 and Figure 7H).

The Radiation Effects Ratio (RER) is the ratio between the biological effect observed with γ- and simGCRsim-IR at the same dose level. Using the same dose level allows the RER to be calculated along the measured dose range, regardless of the model shape or the biological response level (compare the difference between Figure 7C,F,I and the corresponding dose-response models shown in Figure 7A,D,G). The RER values were near one when calculated at an irradiation dose of zero (Table 2), as would be expected, because both irradiation models were estimated at the same beginning control dose point. Beyond the control dose, the RER values fluctuate little; the RER values vary by no more than 0.116 for LVFS, 0.449 for LVEF, and 0.151 for DWS across the dose points measured (Table 2).

## 3. Discussion

How space radiation affects CVD risks remains largely unknown. With NASA’s planned deep-space lunar and Mars missions, it is increasingly necessary to understand ionizing space radiation’s short- and long-term effects on cardiovascular function, in order to estimate the CVD risks during and after long-duration missions. A growing number of epidemiological studies on patients receiving mediastinal RT have helped to delineate the risk of RT-induced CVD, including accelerated atherosclerosis, diastolic dysfunction, pericardial pathologies, and valvulopathies, which can develop years and decades after RT completion [1,2,3,4]. Currently, assessing space-IR-associated CVD risks in astronauts is limited by the statistical constraints of this cohort [26], so that cell and animal studies are needed to help address these knowledge gaps. Numerous such studies using single or fractionated whole-body IR with single HZE ions (16O, 56Fe, 14Si, and 22Ti) have shown HZE IR results in elevated vascular endothelial cell dysfunction, expanded myocardial fibrosis, decreased angiogenesis, altered cardiac compliance, and changed transcriptomes for both cardiomyocytes and endothelial cells (7, 10, 27–30). However, we still lack sufficient knowledge of the degenerative effects of space-relevant radiation on CVD development over the lifespan of an organism.

The purpose of this study was to (1) use non-invasive transthoracic echocardiography to determine the effects of terrestrial (γ) and space radiation (simGCRsim) on LV function, (2) identify CVD spectrum and latency following single whole-body IR, (3) determine whether dose thresholds for γ- and simGCRsim-IR exist, and (4) compare the effects of γ- and simGCR-sim-IR by calculating RBEs and RERs. To explore these lines of inquiry, we irradiated 3-month-old age-matched C57BL/6J wild-type male mice with γ (100 and 200 cGy) and simGCRsim (50 and 100 cGy) IR, and assessed the longitudinal effects of IR on LV function, using echocardiography performed at two acute (14 and 28 days post IR) and three long-term/degenerative (365, 440, and 660 days post IR) time points.

### 3.1. Early Effects

As early as 14 and 28 days post IR, the global systolic function was impaired in both simGCRsim- and γ-IR mice. At 14 days post IR, systolic dysfunction in mice irradiated with 50 cGy simGCRsim IR was associated with reduced stroke volume, LV size, and mass, changes that point to decompensation; however, by 28 days, the LV size and mass recovered, suggesting that the mice had begun compensating. Unlike the mice irradiated with 50 cGy simGCRsim, mice irradiated with either γ-IR dose showed no additional LV structural alterations. As stated earlier, these timepoints (14 and 28 days post IR) approximate “in-flight” time, as ~1 month of mouse life equals 2.9 years of human life, which is also the time estimate for future Mars exploration missions. These findings indicate a significant space-IR-associated decrease in LV systolic function, and particularly combined with space flights’ other confounding factors (especially microgravity), they may be an acute “in-flight” risk that could impede the crew’s physical fitness and performance.

### 3.2. Degenerative Effects

To determine the long-term degenerative effects that single whole-body simGCRsim and γ-IR have on LV function, we assessed mice at 365, 440, and 660 days post IR. At 365 and 440 days post IR, all simGCRsim- and γ-IR WT male mice had diminished global LV systolic function. Although we lack an intermediate timepoint (e.g., 45, 90, or 180 days) to determine whether systolic function recovered beyond 28 days post IR, persistently impaired LV systolic function in both IR groups at later timepoints illustrates the need for early interventions to help recover systolic function. At both 365 and 440 days post IR, the LV structure was not distinctly changed; nevertheless, 50 cGy simGCRsim-IR mice had substantially altered DWS, a load-independent estimate of LV compliance (i.e., LV stiffness), and an index of abnormal systolic and diastolic mechanics [26]. In our prior studies, mice irradiated with single whole-body 50 cGy ^1^H or 15 cGy ^56^Fe-IR showed increased macrophage and monocyte infiltration, and more DNA oxidation at 10 months post IR, despite maintaining cardiac physiology [7]. These findings do not exclude the possibility of subclinical pathological LV impacts.

By 660 days post IR, both IR groups had preserved LV systolic function, suggesting that by this time, the effects of aging may intersect with IR-mediated decreases in LV systolic function. In 50 cGy simGCRsim-IR mice, this preserved LV systolic function was associated with a smaller LV size, a lower mass, and a possible compensation via concentric remodeling (high RWT and low–normal LV mass index). These findings indicate that simGCRsim and γ IR may induce different cardiac remodeling responses.

### 3.3. Cardiac Remodeling

Based on these observed alterations in LV structure and function at degenerative time points, we sought to determine whether simGCRsim- or γ-IR affect the expression of genes involved in the cardiac remodeling processes within the LV. We also evaluated the serum biomarkers of heart failure (BNP), endothelial dysfunction, inflammation, or thrombosis. While serum BNP did not significantly differ in any IR group compared to the ND sham mice across all degenerative time points, transcript levels of BNP and ANP were significantly elevated in 100 cGy and 50 cGy simGCRsim-IR at 660 days. These results may indicate that at this timepoint, mice from both IR groups are experiencing cardiac stress, likely to be pressure overload, as indicated by HFpEF, since both IR groups have preserved LV systolic function at this time point.

At 660 days post IR, transcript levels of *Tgfβ1*, a key mediator of fibrosis, were substantially higher in both IR groups compared to ND sham. Additionally, *Col1a1*, *Mmp9*, and *Mcp1* expression levels were elevated, though not to statistically significant levels, in the LV of 50 cGy simGCRsim-IR mice at 660 days post IR. These results suggest that cardiac fibrosis may be the predominant remodeling process in response to space-type IR exposure. MMP-9, a matrix metalloproteinase involved in degrading the ECM, plays a prominent role in cardiac remodeling after ischemic injury, and is also upregulated during ischemic cardiomyopathy [27,28]. The activation of TGFβ1 via proteases such as MMP-9 promotes TGFβ1-mediated myofibroblast transdifferentiation and the synthesis of ECM (e.g., type I collagen), both of which exacerbate fibrosis [29,30].

Further, at 660 days post IR, the serum of both IR groups contains heightened PAI-1, which inhibits fibrinolysis and is implicated in myocardial fibrosis [31,32]. There is clinical evidence linking increased PAI-1 levels to atherothrombosis [33]. In addition, elevated serum PAI-1 has been associated with hypercoagulable states and impaired fibrinolytic activity in stroke and coronary artery disease, and has recently been connected to major adverse cardiovascular events [31,34]. In mice, PAI-1 protects against cardiac fibrosis during hypertension by inhibiting the urokinase-type plasminogen activator (uPA)-mediated activation of plasminogen (Pg) and limiting plasmin (Pm) generation [25]. 

While *Mmp9* expression was higher, proMMP-9, the zymogen for the active enzyme MMP-9, was significantly lower in both IR groups at 660 days post IR. This may indicate that both IR groups have heightened active protease levels at this time point. proMMP-9 can also be activated via the Pm/MMP-3 cascade [35]. Moreover, inhibiting MMP-9 during the acute stages of cardiovascular disease may provide favorable outcomes, but limiting MMP-9 as the disease progresses may curtail compensatory remodeling and contribute to a progression into HF [30,36].

Fibrosis is also a consequence of inflammation; in both IR groups, the mRNA and serum levels of MCP-1 were elevated, suggesting that inflammation may also drive the remodeling processes after IR. MCP-1 promotes myocardial infiltration via profibrotic leukocyte populations, and it helps to regulate fibroblast behavior [37,38]. MCP-1 serum levels are expanded in patients experiencing heart failure, with preserved ejection fraction (HFpEF); but reduced in patients experiencing heart failure, with reduced ejection fraction (HFrEF) [39,40]. Considering the roles that PAI-1, MMP-9, MCP-1, and TGFβ1 play in CVD, further studies are needed to validate them as targets in simGCRsim-IR-induced cardiac remodeling, and to investigate modulating them as a possible countermeasure for space-IR-induced CVD.

Cardiac beta-myosin heavy chain (βMHC) is an MHC isoform that is associated with lower adenosine triphosphatase activity and filament sliding velocity. βMHC helps to preserve energy during contraction when the overall demand is higher during cardiovascular stress, but it can also depress LV contractile function, and could thus promote hypertrophy and HF development. Interestingly, simCGRsim-IR mice have elevated transcript levels of *βmhc* (365, 440 days post IR); in these mice, hypertrophy may have compensated for the earlier reduction in LV systolic function. Because this may be a space-type IR-specific signature, further work is needed to validate and to elucidate the role of βMHC in space-type IR-induced cardiac remodeling.

Additionally, the transcript levels of *Serca2a*, which is critical in mediating cardiomyocyte contraction and sarcoplasmic reticulum calcium reuptake [18], were also increased in both IR groups at 660 days, although not to statistically significant levels. During HFrEF, SERCA2a activity is reduced, resulting in impaired Ca^2+^ release and subsequently impaired cardiac contraction [18,41]. Since *Serca2a* expression is augmented in both IR types, these mice may be compensating for ancillary remodeling processes by altering ion handling to promote contractility and preservation in LVEF.

Given the observed cardiac fibrosis, the expression and serum levels of TGFβ1 and MCP-1, which are HFpEF signatures [42], and the echocardiography data, it is possible that simGCRsim-IR mice may have phenotypically progressed from the HFrEF to HFpEF phenotype by 660 days post IR. This transition highlights the necessity for a comprehensive, holistic, longitudinal assessment of cardiac physiology via echocardiography. LVEF is the most clinically utilized measure of systolic function in both humans and animal models. Although reduced LVEF directly reflects HFrEF, preserved LVEF (LVEF 55–70% for humans and 60–90% for mice under anesthesia) it does not exclude the possibility of HFpEF. There are significant differences between the underlying causes of HFrEF (ischemic heart disease, tachyarrhythmias, dilated cardiomyopathy, and mitral insufficiency) and those of HFpEF (aging, hypertension, diabetes, obesity, and coronary microvascular disease), and the response to established treatments varies based on the heart failure phenotype [43,44]. Thus, more comprehensive studies of diastolic function via strain echocardiography, and further mechanistic analyses are required to discern the effects of space-IR on systolic and diastolic function [45]. These studies will help to prioritize current cardiovascular therapeutics, such as angiotensin-converting enzyme inhibitors (ACEi) or angiotensin II receptor blockers (ARBs), in mitigating observed alterations in cardiac function and reducing detrimental cardiac remodeling.

### 3.4. Individual Variation

The cardiac response was expected to be relatively homogeneous within the C57BL/6J WT mouse genotypes undergoing each treatment condition; however, we observed considerable inter-animal variability in the LV response across all treatment conditions and time points measured. As such, the fact that the same individuals were not followed longitudinally over time may be a limitation of this study. This individual variability also underscores the need to consider individual baseline (genetic, environmental, and lifestyle) risks when evaluating how spaceflight stressors affect the individual response. Note that the individual cardiac response varies more with increased age and a higher radiation dose.

### 3.5. RBE and RER

The dose response curves for LVEF and LVFS initially drop and then flatten out, a shape that may not be particularly suitable for calculating the RBE. Indeed, this dose-response shape produces results in only a narrow dose-range interval for which the RBE is defined, so that an RBE cannot be calculated for a large portion of the measured dose range (Figure 7). Additionally, many of the dose-response models cross, further contributing to the difficulty in in accurately estimating an RBE. These challenges limit the utility of RBE calculation; nevertheless, the dose-response shape of these cardiac endpoints may be more amenable to an analogous comparative effects calculation, the RER [6]. The RER is the ratio between the biological responses to different IR types for the same dose, so that it remains defined for the entire measured dose range, regardless of the dose-response shape. Dose-response models that cross complicate the accurate estimation of an effects ratio, but the crossing point can be easily identified, as it will be reported as an RER of one. For these reasons, the RER may provide a better estimate for comparing the IR effects in functional cardiac endpoints.

## 4. Materials and Methods

### 4.1. Animal Procedures

Six hundred and sixty male C57BL/6J mice (Jackson Laboratory, Bar Harbor, ME, USA) were shipped directly to the animal facility Brookhaven Laboratory Animal Facility (BLAF) at Brookhaven National Laboratory (BNL, Brookhaven, NY, USA) approximately 1 week before the assigned beamtime. After radiation exposure, mice were housed for 22 months at BLAF to reduce the confounding effect of additional stress caused by repeated transportation to and from a third-party facility for the required 3-month quarantine. Animals were housed in groups of 4–5 per microisolator cage, and they were provided with water and food ad libitum. Both control non-irradiated (ND sham) and irradiated mice were fed commercial mouse chow, referred to as Normal Diet (ND). The experimental design is outlined in Figure 1A. The animal room was kept at a 12:12 h light–dark cycle at 20–22 °C with 30–70% relative humidity.

Animals were monitored at least once a day for any physical or behavioral changes that might indicate distress, discomfort, pain, or injury. Only mice showing the general criteria of poor health and distress, such as rapidly increased heart or respiratory rates, oral or nasal discharge (pus or blood), wound dehiscence, marked swelling, neoplasm(s) greater than 2 cm or ulcerating, an inability to eat or drink, or experiencing a weight loss of greater than 15%, were immediately euthanized using 100% CO_2_ at a 20% air replacement per minute rate, followed by neck dislocation. Any animals found spontaneously dead in their cages were grossly examined, and their carcasses were frozen. Frozen carcasses without significant autolysis were autopsied, and multiple organs (heart, lungs, liver, spleen, kidneys, and intestines) were collected, formalin-fixed, and submitted for routine histology and assessment by a pathologist blindfolded to the study treatment conditions, for postmortem examination only. In this study, mice were sacrificed via exsanguination at 14, 28, 365, 440, and 660 days after irradiation while collecting blood via cardiac puncture and heart tissues for qPCR analysis, histological analysis, or plasma cytokine profiling.

### 4.2. Irradiation Procedures

Animals were exposed to charged heavy ion particle beams at the NASA Space Radiation Laboratory (NSRL) at BNL. Dosimetry studies, including both depth-dose and dose-uniformity measurements, were conducted by beamline physicists. Three-month-old, age-matched male C57BL/6J mice were irradiated with a single full-body dose of ^137^Cs (γ) rays at doses of either 100 cGy or 200 cGy (0.66 MeV), or simGCRsim particles at doses of 50 or 100 cGy (500 MeV/nucleon). Table 3 presents the composition of simGCRsim IR, including ion delivery order, energy, and fraction composition. At the time of simGCRsim exposure, unanesthetized mice were held in 127 × 76.2 × 50.8 mm plastic boxes with 20 holes (3 mm diameter) to provide airflow. Mice were irradiated for ~20 min with simGCRsim IR at the doses noted above. Control sham-treated animals were placed in similar plastic boxes for the equivalent times but were not irradiated. BNL has a ^137^Cs radiation facility suitable for the low LET γ exposures needed to properly calculate the RBEs. All γ-ray exposures were conducted at BNL. The radiation ions, doses, and energies used in these studies were recommended by the Space Radiation Element of NASA’s Human Research Program (HRP).

### 4.3. Echocardiography

Transthoracic echocardiography to assess cardiac structure and function was performed using a GE Vivid E9 with XDclear Cardiac Ultrasound (General Electric Company, Boston, MA, USA) with a GE model i13L probe (General Electric Company). ECHO was performed on all treatment groups at 14, 28, 365, 440, and 660 days after irradiation. Mice were anesthetized with isoflurane (Baxter Healthcare Corporation, Deerfield, IL, USA), and induced at 3% and maintained with 1–2% interprocedurally; hair was removed from the neckline to the mid-chest (using Nair). Mice were then placed supine on a heated table to maintain a core temperature of 37 °C. B- and M-mode images were acquired from a parasternal short-axis view to evaluate the LV ejection fraction (LVEF), LV fractional shortening (LVFS), end-systolic (ESV) and end-diastolic volumes (EDV), LV end-diastolic diameter (LVEDD), LV end-systolic diameter (LVESD), posterior wall thickness at end-diastole (LVPWd) and systole (LVPWs), intraventricular septal thickness (IVSd), left ventricular internal diameter at end diastole (LVIDd), and LVID at end systole (LVIDs), LV mass (1.053 × ((LVIDd + PWthd + IVSd)^3^ − LVIDd^3^)), radial wall strain (DWS; [IVSd + LVPWd]/LVIDd), and relative wall thickness (RWT; ((2 × VPWd)/LVIDd)). Of note, each ECHO parameter is reported as an average of three individual measurements (not repeated measures).

### 4.4. Total RNA Isolation and RT-qPCR Analysis

Total RNA was isolated from heart tissue (LV only) using TRIzol (Invitrogen, #15596018, ThermoFisher Scientific, Waltham, MA, USA) from hearts harvested at 660 days post IR (five samples/group). cDNA was synthesized using the cDNA synthesis kit (Applied Biosystems, #4368814, ThermoFisher Scientific, Waltham, MA, USA) according to the manufacturer’s instructions. Subsequently, real-time quantitative PCR (RT-qPCR) was performed using PerfeCTa SYBR Green FastMix (Quantabio, #95074-012, Beverly, MA, USA) according to the manufacturer’s protocol. mRNA expression was normalized to GAPDH. Primer sequences are listed in Table 4.

### 4.5. Histological Analyses

Hearts harvested at 660 days post IR from ND sham, 50 cGy simGCRsim, and 100 cGy γ-IR mice were transversely cut, fixed in 10% Neutral Phosphate Buffered Formalin (Fisherscientific, #SF100-4, Waltham, MA, USA), and embedded in paraffin blocks. Paraffin sections were cut to 8 µm and subjected to Masson’s trichrome (Sigma-Aldrich, #HT15-1KT, St. Louis, MI, USA) staining to assess the cardiac fibrotic burden. Transverse sections were visualized using a Leica DMi8 microscope. Morphometric histological analyses at basal and midventricular levels (to visualize the LV walls, IVS, and right ventricular walls) were performed using four heart tissue sections taken 80 µm apart from each other. Fibrosis, highlighted in bright blue to contrast with the dark, red-stained myocardium, was quantified using ImageJ. The fraction of the blue stained area was normalized to the total red area. Subepicardial adipose tissue, including the vessel and the pericardium, was subtracted from each slide specimen to avoid fibrosis overestimation. Three animals were studied per treatment group.

### 4.6. Assessment of Plasma Cytokines

The serum levels of the cytokines PAI-1 (total), Pecam-1/sCD31, proMMP-9, sE-Selectin, slCAM-1/sCD54, sP-selectin, Thrombomodulin, and MCP-1 were determined using a Multiplexing LASER Bead Assay (Mouse Cardio Panel 1 and Mouse Cytokine/Chemokine 31-Plex Discovery Assay Array) (Eve Technologies, #MD31, Calgary, AB, Canada). Mice were then anesthetized with isoflurane, 3–4% for induction and 1–2% for maintenance, and blood was collected via cardiac puncture. Blood was kept at room temperature (20–22 °C) for 30 min, and then centrifuged at 1500 rcf for 15 min, after which plasma was carefully extracted. Samples were stored at −80 °C for future use.

### 4.7. Assessment of Hemodynamic Stress

Brain natriuretic peptide (BNP) concentrations were measured from plasma isolated from ND sham, 50 cGy simCGRsim-, and 100 cGy γ-IR mice at three time points (365, 440, and 660 days post IR) using a BNP Enzyme Immunoassay Kit (RayBiotech, #EIAM-BMP, Norcross, GA, USA) according to the manufacturer’s instructions.

### 4.8. Modeling Code and Software

Data visualization, descriptive and inferential statistics, and data analyses were performed with custom-written scripts in Python Version 3.7.6 (Python Software Foundation; http://www.python.org (accessed on 22 June 2020)). Supporting packages/libraries included csv, glob, intertools, matplotlib, numpy, os, pandas, pylab, pynverse, researchpy, scipy, seaborn, and statsmodels. Dose-response curve fitting was performed in the Excel-based program Benchmark Dose Software (BMDS) Version 3.1.2, developed by the U.S. Environmental Protection Agency (EPA) for risk analysis (https://www.epa.gov/bmds (accessed on 22 June 2020)).

### 4.9. Dose-Response Modeling

Dose-response models were fit for the LVEF, LVFS, and DWS datasets (time point, IR type, and dose) that showed statistical significance with ANOVA and post hoc testing. The BMDS Exponential, Hill, Polynomial, Power, and Linear models for continuous endpoints were run. The best fit model for each parameter was chosen by: (i) comparing model fits that showed the lowest AIC (Akaike Information Criterion), (ii) displaying a goodness-of-fit value below α = 0.1, (iii) exhibiting scaled residual values that did not exceed an absolute value of 2, and (iv) visually evaluating the model curves which showed the most biologically relevant shape, given the data resolution.

The Frequentist Exponential degree 4 (Freq. Exp. Deg. 4) model was selected as the best fit to represent the data for LVEF and LVFS:

(1) $$model\left(d\right)=a\times (c-\left(c-1\right)\times e^{-b*d})$$

where *a* is the control response (intercept), *b* is the slope, *c* is the asymptote term, and *d* is the dose. Parameter constraints include: *a* > 0, *b* > 0, *c* > 1 for responses increasing with dose, 0 < *c* < 1 for responses decreasing with dose, and 1 ≤ *n* ≤ 18.

The linear model was selected as the best fit to represent the data for DWS:

(2) $$model\left(d\right)=g+\beta_{1}\times d$$

where *g* is the control (intercept), *β*_1_ is the polynomial coefficient, and *d* is the dose.

To produce dose-response models that could directly approximate the disease incidence, LVEF and LVFS were dichotomized into categories of “normal” and “diseased” cardiac function. Parameters categorized as “diseased” represent either a significantly diminished capacity for physical activity or heart failure in development. “Normal” or “diseased” cutoffs were assigned for the LVFS and LVEF parameters. LVFS was defined as being “normal” within a range of 40–100%, and as “diseased” for LVFS below 40%. LVEF was defined as “normal” within a range of 50–100%, and as “diseased” for LVEF below 50%, representing systolic dysfunction. Dose-response models were fit to the dichotomized data for each dataset that showed statistical significance with ANOVA and post hoc testing. Dichotomized dose-response models were analyzed but were not used in subsequent analyses (see the reasoning given in the Results section, “*Dose-response models with dichotomized data*”).

### 4.10. RBE and RER Calculations

The selected BMDS dose-response models were used to calculate the RBEs and RERs to compare the γ- and simGCRsim-IR functional cardiac responses. Only datasets that showed a statistical significance between the dose cohorts with hypothesis testing were modeled in BMDS and were used to calculate the RBE and RER. Lower LVFS and LVEF values (measured in percentage) in this echocardiography data indicate more adverse outcomes; the complement of the percentage was taken as the response value before the comparison ratios were calculated. This adjustment allowed the RBE and the RER to be directly interpreted as surrogate markers of risk.

The RBE is the ratio between the radiation doses for a given biological response level. As the response is the output of the original BMDS model, we used the model inverse. This converted the BMDS model, which is written as a function of the dose that reports the response, to a model configured as a function of the response that reports the dose.

With the previous adjustments, RBE was calculated as follows:

(3) $$RBE=\frac{D_{\gamma }}{D_{I}}$$

where *D_γ_* is the dose for γ-IR and *D_I_* is the dose of simGCRsim-IR at a given response. The *RBE* was reported as a function of the simGCRsim IR dose. 

The RER calculates the ratio of the responses observed between the compared IR types for a given dose [6]. As the BMDS models are a function of dose and report the biological response, they could be used to calculate the RER directly without adjustment. The *RER* was calculated as follows:

(4) $$RER=\frac{R_{\gamma }}{R_{I}}$$

where *R_γ_* is the biological response to γ IR and *R_I_* is the biological response to simGCRsim-IR for a given dose. 

### 4.11. Statistics

#### 4.11.1. For Echocardiography and Serum Markers

The ECHO parameters for ND sham and both IR groups were compared using one-way ANOVA with the Tukey post hoc test, given that the data passed the assumptions required for normality (Shapiro-Wilk) and heteroscedasticity (Brown-Forsythe). qPCR and serum biomarkers in the ND sham and both IR groups were compared using the Kruskal-Wallis test with Dunn’s correction for multiple comparisons. All analyses were performed using GraphPad Prism 8, version 8.4.3 (GraphPad Software, Inc., La Jolla, CA, USA). Data are expressed as mean ± SEM, except in box plots where the whiskers extend from the minimum to the maximum. Differences were considered as being statistically significant at *p* < 0.05.

#### 4.11.2. For Modeling

Normal data distribution was determined with an array of data visualizations and tests for normality (the Kolmogorov-Smirnov test and the Shapiro-Wilk test), skew, and kurtosis. Homoscedasticity, or equality of variance, was confirmed with Levene’s Test. With these prerequisite assumptions met, a one-way analysis of variance (ANOVA) was run to determine the statistical significance between doses for a given mouse strain, IR type, and time point. ANOVA hypothesis testing was followed up with Tukey’s HSD (honestly significant difference) post hoc test for three datasets that showed significance with ANOVA to distinguish the significance between the control and the IR cohorts.

### 4.12. Study Approval

All animal procedures were performed in accordance with the standards of the Guide for the Care and Use of Laboratory Animals for the National Institutes of Health, and approved by the Animal Care and Use Committees at Brookhaven National Laboratory (BNL) (Upton, NY, USA) (BNL IACUC Protocol #502) and the Icahn School of Medicine at Mount Sinai (New York, NY, USA) (ISMMS IACUC Protocol #2019-0017).

## 5. Conclusions

Our work is the first to assess in mice the lifetime longitudinal effects of simGCRsim-IR at doses of 50 and 100 cGy, and of γ-IR at doses of 100 and 200 cGy, on LV function and structure, using echocardiography, serum biomarkers, and cardiac tissue structure alterations. Our findings can be summarized as follows: (1) LV systolic function is impaired in simGCRsim (50 and 100 cGy) and γ-IR (100 and 200 cGy) wild-type C57BL/6J male mice as early as 14 and 28 days post IR, as well as 365 and 440 days post IR; (2) at 660 days post IR, simGCRsim-IR male mice appear to have developed a HFpEF cardiac phenotype, though diastolic dysfunction cannot be ruled out, while cardiac function is recovered in γ-IR mice; (3) cardiac function alterations at 660 days post IR appear to be associated with an increased expression of markers of cardiac fibrosis (*Tgfβ1* and *Mmp9*), inflammation (*Mcp1*), and hypertrophy (*βmhc*), results that suggest that simGCRsim-IR may dynamically affect the cardiac remodeling processes throughout a lifetime; and (4) no clear dose-response was observed. Further studies to assess sex differences and the use of translational models such as minipigs or non-human primates are needed to better evaluate how varied doses of space-IR affect CVD risks. Ongoing studies in our lab are exploring the acute and degenerative effects of simGCRsim- and γ-IR on female C57BL/6J mice. Finally, our current findings do not exclude the possibility of elevated acute or degenerative CVD risks at lower doses of space-type IR, and/or when combined with other space travel-associated stressors, such as microgravity; these are questions that require further investigation.

## Figures and Tables

**Table 1:** RBE values at the minimum and maximum simGCRsim dose range where the calculation was defined. The models for LVFS on day 440 and for DWS were more linear in shape, and therefore showed an RBE and maximum simGCRsim dose near the theoretical maximum response level of 100%.

Response	Days after IR	Min simGCRsim Dose (cGy)	RBE	Max simGCRsim Dose (cGy)	RBE
LVEF (%)	14	5.97665465	0.126348	107.954502	0.33423
	28	4.512451222	0.842589	104.6594033	1.825615
	365	0.2615792	2.46268	8.01562382	3.802952
	440	2.212052131	19.74135	100.599105	2.295173
LVFS (%)	14	2.977494895	0.193363	104.3445831	0.306601
	28	5.689671903	0.840422	103.3125764	2.979034
	365	0.185973405	1.087121	19.83534394	1.853151
	440	8.497120528	7.655587	1737.15217	2.221847
DWS	365	5.271755497	0.438695	459.969739	2.807636
	440	5.830303739	6.525775	621.9755207	2.839298

**Table 2:** RER values at the measured gamma and simGCRsim dose levels.

		Radiation Dose (cGy)				
Response	Day after IR	0	50	100	150	200
LVEF (%)	14	0.990423	1.354822	1.055008	0.870207	0.746942
	28	0.982736	1.060224	0.967893	0.861646	0.771131
	365	1.000013	0.911371	0.915443	0.915489	0.91549
	440	0.951101	0.814465	0.847583	0.90196	0.958549
LVFS (%)	14	0.999928	1.084308	1.015748	0.983877	0.968795
	28	0.993236	1.020032	0.982412	0.934373	0.890226
	365	1.000009	0.968722	0.967345	0.967311	0.96731
	440	0.975545	0.947676	0.926543	0.910477	0.898302
DWS	365	1.006697	0.96099	0.921255	0.886393	0.85556
	440	0.992132	0.961376	0.933461	0.908008	0.884706

**Table 3:** Composition of simGCRsim irradiation used in this study, with ions listed in delivery order. Delivering 6 beams and 5 ions for simGCRsim requires about 20 min.

Ion	Energy (MeV/n)	Fraction (%)
^1^H	1000	35
^28^Si	600	1
^4^He	250	18
^16^O	350	6
^56^Fe	600	1
^1^H	1000	39

**Table 4:** Primer list for genes involved in cardiac remodeling.

Gene	Primers (5′–3′)
*Tgfβ1*	F-CCTGCAAGACCATCGACATGGAG R-GGTCGCGGGTGCTGTTGTA
*Col1a1*	F-CTGGCAAGAAGGGAGATGA R-CACCATCCAAACCACTGAAA
*Col3a1*	F-GATGGAAACCCTGGATCAGA
*Ncx*	F-TTTGCCTTCGTCCCACCTAC R-AACGGCAGTCACGGAATCTT
*Serca2a*	F-ACGCCTGCAACTCGGTCATA R-ATGTCCGGCTTGGCTTGTTT
*Tnfα*	F-CAAGTGGAGGAGCAGCTGGA R-CTGACGGTGTGGGTGAGGAG
*Mcp1*	F-TGCAGGTCCCTGTCATGCTT R-TCTTTGGGACACCTGCTGCT
*Mmp9*	F-CGCTCATGTACCCGCTGTAT R-CCGTGGGAGGTATAGTGGGA
*Gals3*	F-ACAGTCAGCCTTCCCCTTTG R-GTTAGCGCTGGTGAGGGTTA
*βmhc*	F-ACTGTCAACACTAAGAGGGTCA R-TTGGATGATTTGATCTTCCAGGG
*Anp*	F-GCTTCCAGGCCATATTGGAG R-GGGGGCATGACCTCATCTT
*Bnp*	F-CTGGAAGTCCTAGCCAGTC R-TTTTCTCTTATCAGCTCCAGCA
*Gapdh*	F-GTGAAGGTCGGTGTGAACG R-TCGTTGATGGCAACAATCTC

## Data Availability

All data generated or analyzed during this study are included in this published article and its Supplementary Information Files. Any additional datasets used and/or analyzed during the current study are available from the corresponding author on reasonable request.

## Supplementary Materials

The supporting information can be downloaded at: https://www.mdpi.com/article/10.3390/ijms24065451/s1.

## References

[1] Darby S.C., Ewertz M., McGale P., Bennet A.M., Blom-Goldman U., Bronnum D., Correa C., Cutter D., Gagliardi G., Gigante B. (2013). Risk of ischemic heart disease in women after radiotherapy for breast cancer. N. Engl. J. Med..

[2] Cutter D.J., Schaapveld M., Darby S.C., Hauptmann M., van Nimwegen F.A., Krol A.D., Janus C.P., van Leeuwen F.E., Aleman B.M. (2015). Risk of valvular heart disease after treatment for Hodgkin lymphoma. J. Natl. Cancer Inst..

[3] Wang H., Wei J., Zheng Q., Meng L., Xin Y., Yin X., Jiang X. (2019). Radiation-induced heart disease: A review of classification, mechanism and prevention. Int. J. Biol. Sci..

[4] Raghunathan D., Khilji M.I., Hassan S.A., Yusuf S.W. (2017). Radiation-Induced Cardiovascular Disease. Curr. Atheroscler. Rep..

[5] Barcellos-Hoff M.H., Mao J.H. (2016). HZE Radiation Non-Targeted Effects on the Microenvironment That Mediate Mammary Carcinogenesis. Front. Oncol..

[6] Shuryak I., Fornace A.J., Datta K., Suman S., Kumar S., Sachs R.K., Brenner D.J. (2017). Scaling Human Cancer Risks from Low LET to High LET when Dose-Effect Relationships are Complex. Radiat. Res..

[7] Yan X., Sasi S.P., Gee H., Lee J., Yang Y., Mehrzad R., Onufrak J., Song J., Enderling H., Agarwal A. (2014). Cardiovascular risks associated with low dose ionizing particle radiation. PLoS ONE.

[8] Sasi S.P., Yan X., Zuriaga-Herrero M., Gee H., Lee J., Mehrzad R., Song J., Onufrak J., Morgan J., Enderling H. (2017). Different Sequences of Fractionated Low-Dose Proton and Single Iron-Radiation-Induced Divergent Biological Responses in the Heart. Radiat. Res..

[9] Tungjai M., Whorton E.B., Rithidech K.N. (2013). Persistence of apoptosis and inflammatory responses in the heart and bone marrow of mice following whole-body exposure to ^28^Silicon (^28^Si) ions. Radiat. Environ. Biophys..

[10] Garikipati V.N.S., Arakelyan A., Blakely E.A., Chang P.Y., Truongcao M.M., Cimini M., Malaredy V., Bajpai A., Addya S., Bisserier M. (2021). Long-Term Effects of Very Low Dose Particle Radiation on Gene Expression in the Heart: Degenerative Disease Risks. Cells.

[11] Lindsey M.L., Kassiri Z., Virag J.A.I., de Castro Bras L.E., Scherrer-Crosbie M. (2018). Guidelines for measuring cardiac physiology in mice. Am. J. Physiol. Heart Circ. Physiol..

[12] Hsu S., Fang J.C., Borlaug B.A. (2022). Hemodynamics for the Heart Failure Clinician: A State-of-the-Art Review. J. Card. Fail..

[13] Gardin J.M., Siri F.M., Kitsis R.N., Edwards J.G., Leinwand L.A. (1995). Echocardiographic assessment of left ventricular mass and systolic function in mice. Circ. Res..

[14] Scherrer-Crosbie M., Thibault H.B. (2008). Echocardiography in translational research: Of mice and men. J. Am. Soc. Echocardiogr..

[15] Takeda Y., Sakata Y., Higashimori M., Mano T., Nishio M., Ohtani T., Hori M., Masuyama T., Kaneko M., Yamamoto K. (2009). Noninvasive assessment of wall distensibility with the evaluation of diastolic epicardial movement. J. Card. Fail..

[16] Li V.W., Cheuk D.K., Cheng F.W., Yang J.Y., Yau J.P., Ho K.K., Li C.K., Li R.C., Yuen H.L., Ling A.S. (2017). Myocardial stiffness as assessed by diastolic wall strain in adult survivors of childhood leukaemias with preserved left ventricular ejection fraction. Eur. Heart J. Cardiovasc. Imaging.

[17] Frangogiannis N.G. (2019). Cardiac fibrosis: Cell biological mechanisms, molecular pathways and therapeutic opportunities. Mol. Asp. Med..

[18] Liu Z., Ni J., Li L., Sarhene M., Guo R., Bian X., Liu X., Fan G. (2020). SERCA2a: A key protein in the Ca^2+^ cycle of the heart failure. Heart Fail. Rev..

[19] Niu J., Kolattukudy P.E. (2009). Role of MCP-1 in cardiovascular disease: Molecular mechanisms and clinical implications. Clin. Sci..

[20] Krenz M., Robbins J. (2004). Impact of beta-myosin heavy chain expression on cardiac function during stress. J. Am. Coll. Cardiol..

[21] Nakagawa Y., Nishikimi T., Kuwahara K. (2019). Atrial and brain natriuretic peptides: Hormones secreted from the heart. Peptides.

[22] Gurbel P.A., Serebruany V.L., Shustov A.R., Dalesandro M., Gumbs C.I., Grablutz L.B., Bahr R.D., Ohman E.M., Topol E.J. (1998). Increased baseline levels of platelet P-selectin, and platelet-endothelial cell adhesion molecule-1 in patients with acute myocardial infarction as predictors of unsuccessful thrombolysis. Coron. Artery Dis..

[23] Serebruany V.L., Gurbel P.A. (1999). Effect of thrombolytic therapy on platelet expression and plasma concentration of PECAM-1 (CD31) in patients with acute myocardial infarction. Arterioscler. Thromb. Vasc. Biol..

[24] Xu Z., Castellino F.J., Ploplis V.A. (2010). Plasminogen activator inhibitor-1 (PAI-1) is cardioprotective in mice by maintaining microvascular integrity and cardiac architecture. Blood.

[25] Gupta K.K., Donahue D.L., Sandoval-Cooper M.J., Castellino F.J., Ploplis V.A. (2017). Plasminogen Activator Inhibitor-1 Protects Mice Against Cardiac Fibrosis by Inhibiting Urokinase-type Plasminogen Activator-mediated Plasminogen Activation. Sci. Rep..

[26] Ohtani T., Mohammed S.F., Yamamoto K., Dunlay S.M., Weston S.A., Sakata Y., Rodeheffer R.J., Roger V.L., Redfield M.M. (2012). Diastolic stiffness as assessed by diastolic wall strain is associated with adverse remodelling and poor outcomes in heart failure with preserved ejection fraction. Eur. Heart J..

[27] Romanic A.M., Harrison S.M., Bao W., Burns-Kurtis C.L., Pickering S., Gu J., Grau E., Mao J., Sathe G.M., Ohlstein E.H. (2002). Myocardial protection from ischemia/reperfusion injury by targeted deletion of matrix metalloproteinase-9. Cardiovasc. Res..

[28] Halade G.V., Jin Y.F., Lindsey M.L. (2013). Matrix metalloproteinase (MMP)-9: A proximal biomarker for cardiac remodeling and a distal biomarker for inflammation. Pharmacol. Ther..

[29] Yu Q., Stamenkovic I. (2000). Cell surface-localized matrix metalloproteinase-9 proteolytically activates TGF-beta and promotes tumor invasion and angiogenesis. Genes Dev..

[30] Morishita T., Uzui H., Mitsuke Y., Amaya N., Kaseno K., Ishida K., Fukuoka Y., Ikeda H., Tama N., Yamazaki T. (2017). Association between matrix metalloproteinase-9 and worsening heart failure events in patients with chronic heart failure. ESC Heart Fail..

[31] Lucore C.L., Sobel B.E. (1988). Interactions of tissue-type plasminogen activator with plasma inhibitors and their pharmacologic implications. Circulation.

[32] Ghosh A.K., Vaughan D.E. (2012). PAI-1 in tissue fibrosis. J. Cell. Physiol..

[33] Aso Y. (2007). Plasminogen activator inhibitor (PAI)-1 in vascular inflammation and thrombosis. Front. Biosci..

[34] Jung R.G., Motazedian P., Ramirez F.D., Simard T., Di Santo P., Visintini S., Faraz M.A., Labinaz A., Jung Y., Hibbert B. (2018). Association between plasminogen activator inhibitor-1 and cardiovascular events: A systematic review and meta-analysis. Thromb. J..

[35] Hahn-Dantona E., Ramos-DeSimone N., Sipley J., Nagase H., French D.L., Quigley J.P. (1999). Activation of proMMP-9 by a plasmin/MMP-3 cascade in a tumor cell model. Regulation by tissue inhibitors of metalloproteinases. Ann. N. Y. Acad. Sci..

[36] Radosinska J., Barancik M., Vrbjar N. (2017). Heart failure and role of circulating MMP-2 and MMP-9. Panminerva Med..

[37] Sakai N., Wada T., Furuichi K., Shimizu K., Kokubo S., Hara A., Yamahana J., Okumura T., Matsushima K., Yokoyama H. (2006). MCP-1/CCR2-dependent loop for fibrogenesis in human peripheral CD14-positive monocytes. J. Leukoc. Biol..

[38] Lehmann M.H., Kuhnert H., Muller S., Sigusch H.H. (1998). Monocyte chemoattractant protein 1 (MCP-1) gene expression in dilated cardiomyopathy. Cytokine.

[39] Boyle A.J., Yeghiazarians Y., Shih H., Hwang J., Ye J., Sievers R., Zheng D., Palasubramaniam J., Palasubramaniam D., Karschimkus C. (2011). Myocardial production and release of MCP-1 and SDF-1 following myocardial infarction: Differences between mice and man. J. Transl. Med..

[40] Kobayashi M., Nakamura K., Kusano K.F., Nakamura Y., Ohta-Ogo K., Nagase S., Sakuragi S., Ohe T. (2008). Expression of monocyte chemoattractant protein-1 in idiopathic dilated cardiomyopathy. Int. J. Cardiol..

[41] Limas C.J., Olivari M.T., Goldenberg I.F., Levine T.B., Benditt D.G., Simon A. (1987). Calcium uptake by cardiac sarcoplasmic reticulum in human dilated cardiomyopathy. Cardiovasc. Res..

[42] Simmonds S.J., Cuijpers I., Heymans S., Jones E.A.V. (2020). Cellular and Molecular Differences between HFpEF and HFrEF: A Step Ahead in an Improved Pathological Understanding. Cells.

[43] Guazzi M., Ghio S., Adir Y. (2020). Pulmonary Hypertension in HFpEF and HFrEF: JACC Review Topic of the Week. J. Am. Coll. Cardiol..

[44] Dzhioeva O., Belyavskiy E. (2020). Diagnosis and Management of Patients with Heart Failure with Preserved Ejection Fraction (HFpEF): Current Perspectives and Recommendations. Ther. Clin. Risk Manag..

[45] de Lucia C., Wallner M., Eaton D.M., Zhao H., Houser S.R., Koch W.J. (2019). Echocardiographic Strain Analysis for the Early Detection of Left Ventricular Systolic/Diastolic Dysfunction and Dyssynchrony in a Mouse Model of Physiological Aging. J. Gerontol. A Biol. Sci. Med. Sci..

