# Supplementary material for: Lifetime Evaluation of Left Ventricular Structure and Function in Male C57BL/6J Mice after Gamma and Space-Type Radiation Exposure
Source: Int J Mol Sci. 2023 Mar 13;24(6):5451. doi: 10.3390/ijms24065451 (PMC10049327; doi:10.3390/ijms24065451)
Supplement: Supplementary file 1 [file ijms-24-05451-s001.zip › ijms-2197865-supplementary.pdf]

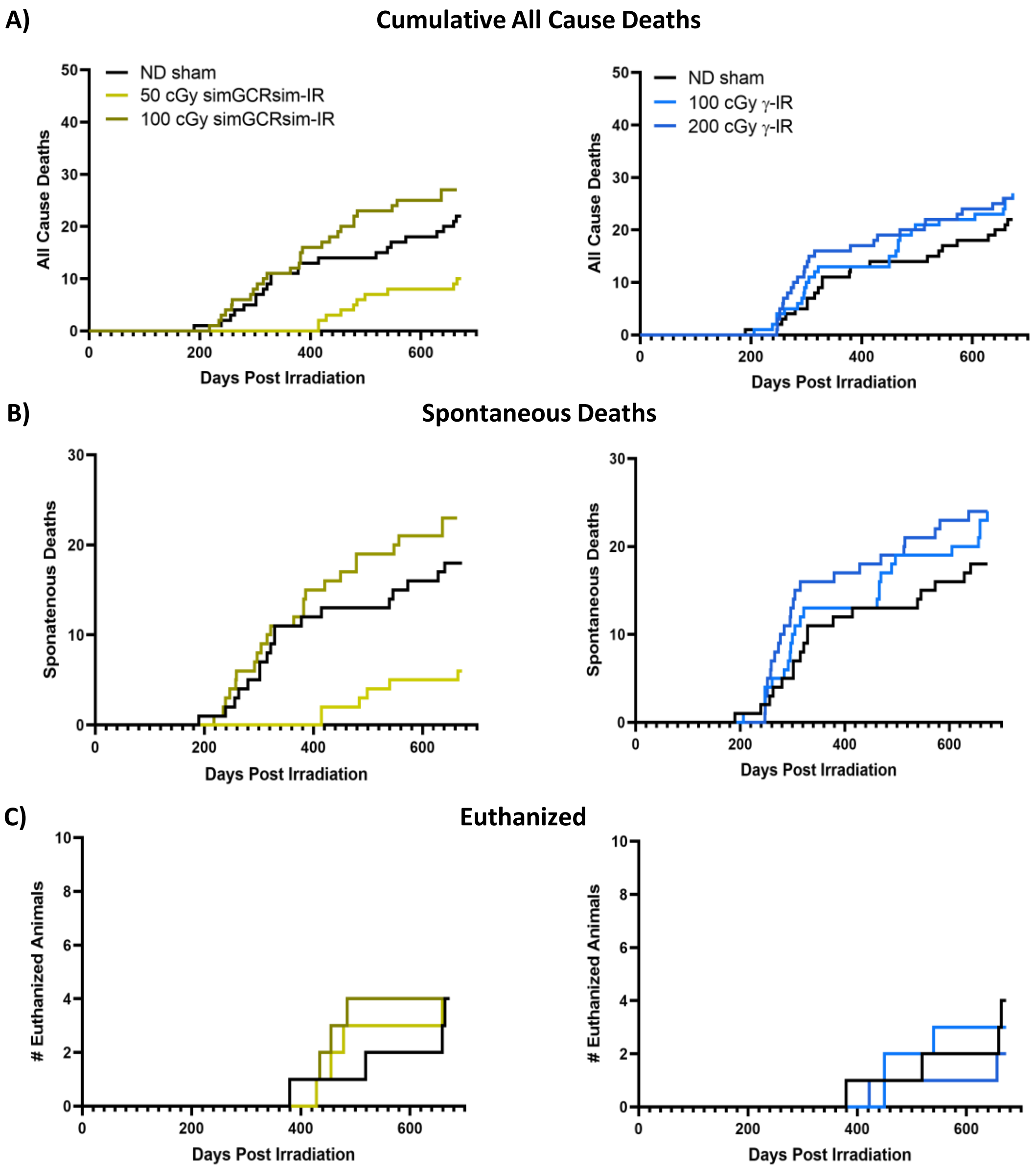

Supplementary Figure S1. Mortality in C57BL/6J male mice irradiated with gamma and simGCRsim irradiation. Graphs depicting the total number of (A) all-cause deaths, including (B) spontaneous deaths, defined as mice found dead in cages, and (C) animals euthanized during the course of experiments for adverse health effects.

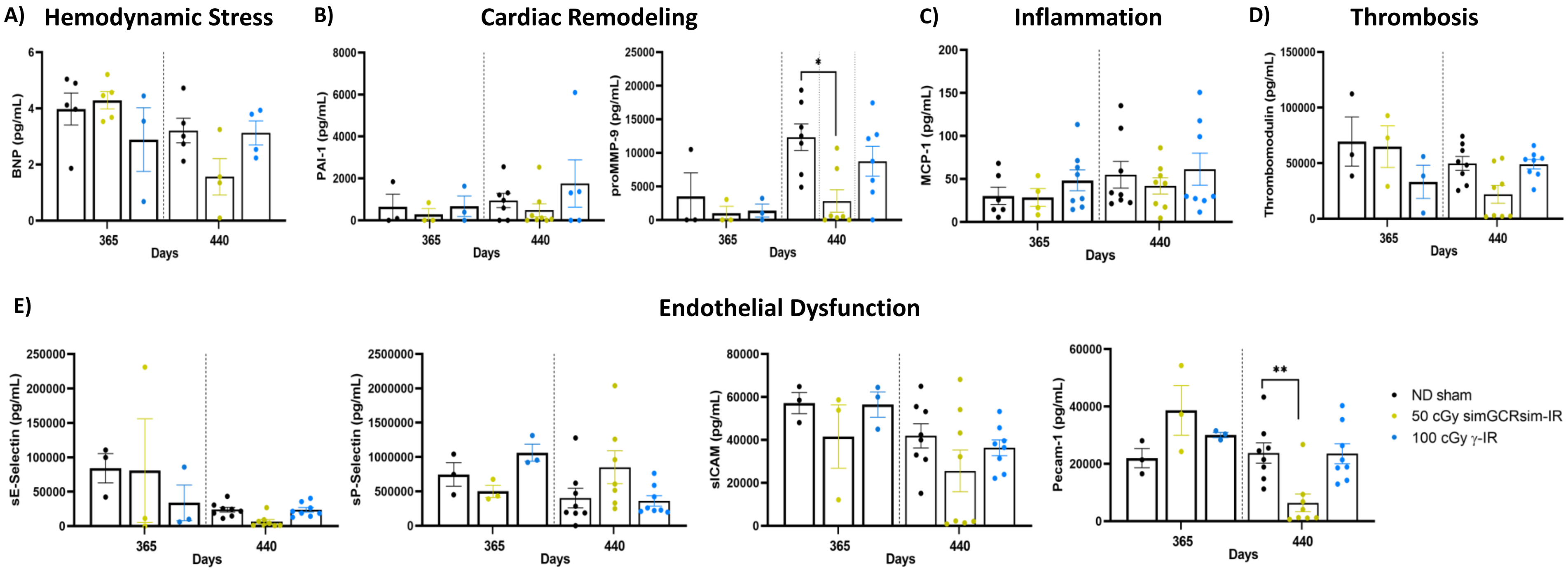

Supplementary Figure S2. Select cardiac serum markers assessed 365 and 440 days after gamma and simGCRsim irradiation. Peripheral blood serum was processed using standard ELISA to assess for serum levels of (A) brain natriuretic peptide (BNP). Remaining samples were used for Multiplex assay to assess for markers of (B) cardiac remodeling: plasminogen activator inhibitor-1 (PAI-1) and pro-matrix metalloprotease-9 (proMMP-9), (C) inflammation: Monocyte Chemoattractant Protein-1 (MCP-1), (D) Thrombosis: thrombomodulin, and (E) endothelial dysfunction: sE-Selectin, sP-Selectin, sICAM, PECAM-1. All values are reported as pg/mL as mean ± SEM. N=3-8 animals/treatment group; p-values were calculated using Kruskal-Wallis, *p <0.05.

	No-IR	simGCRsim-IR		γ-IR	
	ND-fed	50 cGy	100 cGy	100 cGy	200 cGy
Myocardial injury/heart failure/vascular injury					
Mild to moderate multifocal myocardial replacement fibrosis with myocardial mineralization	1	-	-	1	-
Moderate, multifocal acute degeneratoion of myocardiym with mild multifocal mineralization	1	-	-	-	-
Severe, multifocally extensive, myocardial degeneration, fibrosis and mineralization	-	-	-	1	-
Multifocal subepicardial myocardial mineratization	1	-	-	-	-
Severe, multifocal vascular mineralization	-	-	-	1	-
Extensive intimal mineratization of aorta	1	-	-	-	-
Mild, multifocal myocardial fibrosis, disarray and hypertrophy	-	-	-	1	-
Acute necrotizing myocarditis	1	-	-	-	-
Cancer					
Histiocytic sarcoma	-	-	-	1	-
Metastatic Lymphoma	-	-	-	1	-
Leukemia	-	1	-	-	-
Sepsis and/or infectious disease/inflammation					
Acute interstitial pneumonia with intravascular bacteria	3	-	-	-	-
Intrahistiocytic bacteria in spleen	-	1	-	-	-
Acute multifocal hepatitis with intralesional bacteria	1	-	-	-	-
Intravascular bacteria in heart	1	-	-	-	-
Vascular neutrophilia	-	-	-	-	1
Possible toxic injury					
Severe diffuse vacuolar degeneration in liver	2	-	-	-	-
Inflammation					
Severe lymphoid atrophy	-	-	-	1	-
Multifocal microthrombi with perivascular lymphoplasmacytic vasculitis	1	-	-	-	-
Renal					
Marked,chronic glomerulonephropathy	-	-	-	1	-
Hepatic					
Marked, multifocal hepatic necrosis and infarcts	-	1	-	-	-
Hepatocellular adenoma	-	-	1	-	-
Hepatocellular hyperplasia with cystic degeneration, necrosis	-	-	1	-	-
Other					
Unknown Cause	2	-	-	2	-
Rule out post-mortem leakage in lungs	3	2	2	2	1

Supplementary Table S1. Autopsy data from mice found spontaneously dead or euthanized during the course of the longitudinal lifetime experiment.
